# Old, Nonagenarians, and Centenarians in Cilento, Italy and the Association of Lifespan with the Level of Some Physicochemical Elements in Tap Drinking Water

**DOI:** 10.3390/nu15010218

**Published:** 2023-01-01

**Authors:** Silvana Mirella Aliberti, Richard H. W. Funk, Elena Ciaglia, Joseph Gonnella, Aldo Giudice, Carmine Vecchione, Annibale Alessandro Puca, Mario Capunzo

**Affiliations:** 1 Department of Medicine, Surgery and Dentistry “Scuola Medica Salernitana”, University of Salerno, 84081 Salerno, Italy; 2 Medical Faculty Carl Gustav Carus, Institute of Anatomy, University of Technology (TU) Dresden, 01307 Dresden, Germany; 3 Center for Medical Research in Medical Education and Health Care, Sidney Kimmel Medical College, Thomas Jefferson University, Philadelphia, PA 19107, USA; 4 Epidemiology Unit, Istituto Nazionale Tumori IRCCS, “Fondazione G. Pascale”, 80131 Naples, Italy; 5 Vascular Physiopathology Unit, Department of Angio-Cardio-Neurology, IRCCS Neuromed, 86077 Pozzilli, Italy; 6 Cardiovascular Research Unit, IRCCS MultiMedica, 20138 Milan, Italy; 7 Complex Operational Unit Health Hygiene, University Hospital “San Giovanni di Dio e Ruggi d’Aragona”, 84131 Salerno, Italy

**Keywords:** longevity, old population, centenarians, physicochemical elements, tap drinking water, healthy living, Cilento region

## Abstract

Longevity, as a complex life-history trait, shares an ontogenetic relationship with other quantitative traits, such as epigenetic and environmental factors. Therefore, it is important to identify environmental factors that may modify the epigenome to establish healthy aging. This study explored the association between tap drinking water and longevity in Cilento, Italy, to understand whether trace elements in local drinking water may have an influence on old, nonagenarian, and centenarian people and promote their health and longevity. Data on population and water sources were collected through the National Demographic Statistics, the Cilento Municipal Archives, and the Cilento Integrated Water Service. Ordinary least squares (OLS) regression and a geographically weight regression (GWR) model were used to study the spatial relationship between the explanatory and outcome variables of longevity. The results of the study showed that the prevalence of longevity is concentrated in the central, northern and southeastern areas of the territory and that some trace elements present in tap water may contribute to local longevity in Cilento. Specifically, all Cilento municipalities had alkaline tap water, and the municipalities with the highest longevity concentrations had higher alkalinity levels than the other municipalities, soft to medium-hard water hardness, an amount of total dissolved solids equivalent to the level of excellent water, lower amounts of sodium, adequate iron concentration, and adequate dietary intake of manganese per day.

## 1. Introduction

There has long been a global desire to be able to achieve greater longevity, not only in terms of overall lifespan, but especially regarding the possibility of increasing the healthy part of the life. To activate the strategies necessary to achieve this goal, a whole range of factors and the complex interactions among them must be considered. Many studies have shown that longevity is closely linked to genetic factors [1,2,3,4] and macro-environmental (climate, land, water) [5,6] and micro-environmental (lifestyle, social and community networks, socio-economic conditions) [7,8,9] factors.

Epigenetics (a term coined in 1942 by Waddington) stufies the interconnected concatenations of factors that take place in the space between the genotype and phenotype. Environmental factors can trigger a series of changes in physiological developmental patterns through a process termed developmental plasticity, causing an individual to produce alternative phenotypes throughout life, and these changes can have beneficial effects on health [10]. 

In recent years, among the macro-environmental factors, special attention has been paid to the relationship between drinking water and physical health [11]. Water is essential to life [12]. On the other hand, in the human body, water is linked to almost all interfaces between internal structures, including ions, proteins, membranes, and organelles, and, due to its special physicochemical properties, many vital functions can take place regularly and create benefits [13]. Drinking water helps eliminate toxins and waste substances, normalizes body temperature, detoxifies and purifies the body, facilitates the transport of nutrients to cells, aids in digestion, and combats fatigue and tiredness [14]. According to the World Health Organization (WHO) [15], drinking water contains beneficial elements such as fluoride (F), iron (Fe), manganese (Mn), nickel (Ni), zinc (Zn), etc., which are essential for the growth of the human body and living organisms. Essential mineral elements are significantly assimilated through drinking water [15,16] and, depending on its physicochemical quality, may have a beneficial influence on, or cause risks to, health. 

Several studies have examined potential human health risks from As, F and heavy metals in low-quality groundwater [17,18,19]. Some research has considered sodium content in water deleterious because it potentially induces hypertension, although chloride concentration may also contribute to this [20,21,22]. According to a study conducted in 76 municipalities in central Sweden on regional differences in cardiovascular disease mortality, a significant gradient was found between western areas with high mortality and soft water and eastern areas with low mortality and hard water [23]. Dore et al. [24] confirm the beneficial role of water high in calcium and bicarbonate against coronary heart disease. Other studies in animal models have shown positive effects on improved body weight and offspring development [25,26] with the use of alkaline water. In addition, several biological effects have been documented, such as antidiabetic and antioxidant actions [27], DNA-protective effects [28], and growth-promoting activities [26]. According to Sengupta [12] hard water is considered an important etiological factor, causing many diseases such as cardiovascular problems, diabetes, reproductive failure, neuronal diseases, etc. 

The relationship between the characteristics of major drinking water elements in some typical longevity areas has been demonstrated by a number of studies [29,30,31].

In agreement with Hao et al. [29], a positive correlation was found between daily intake of copper (Cu), selenium (Se) and zinc (Zn) from food and water and indices of aging and longevity, and a negative correlation between lead (Pb) and indices of longevity. Liu et al. [32] found that moderate water hardness and higher strontium concentration in drinking water characterize municipalities with longevity compared with those with non-longevity. Research by Moretti et al. [33] using a short-lived invertebrate model (i.e., fruit flies) showed that the consumption of very low dietary doses of nitrite can increase the lifespan. However, the results were only evident in females, suggesting a possible sex-specific effect of this anion. The research results of Carvalho et al. [34] suggest the that long-term consumption of inorganic nitrate is safe, does not increase the risk of death and does not affect the lifespan of animals, regardless of gender. 

Therefore, the quality of drinking water from different natural environments could have a direct impact on local human health and longevity [15,31,32].

Despite numerous studies on the topic of longevity and correlations with various environmental factors, to the best of our knowledge, no research conducted in Cilento or other Italian regions has investigated the possible relationship between longevity and trace elements in drinking water at the municipal level. Therefore, we hypothesized that some physicochemical properties of drinking water may be protective factors for longevity.

The purpose of this study was to explore the association between tap drinking water and longevity in Cilento and to understand whether trace elements in drinking water can have a direct influence on local health and longevity and improve the living standards of the old, nonagenarians and centenarians.

## 2. Materials and Methods

### 2.1. Description of the Study Area: The Cilento Region

Geographically, Cilento is a subregion of the Campania region of southern Italy, in the territory of the province of Salerno, and has been declared a UNESCO World Heritage Site. The territory of Cilento covers an area of about 490,000 hectares and includes 102 municipalities. From the environmental point of view, it is a heterogeneous territory, characterized by the integration of different landscape types, including coastal, hilly and mountainous areas [5] and mouths of important waterways, and the morphological and climatic variability creates a potential not easily found in other areas of the Italian peninsula [35]. The geological nature of the rocks that make up the Cilento is twofold: the “Cilento flysch” (dense stratification of rocks) toward the northwestern coast, and the “limestone rocks” (generators of karst phenomena) that make up the inland (Alburno-Cervati) and southern mountain complexes of the Cilento and Vallo di Diano National Park [35]. Karst phenomena are one of the determinants of the lithological characteristics of Cilento waters [35,36], along with soil leaching and atmospheric precipitation [32]. It should be noted that Cilento has a large number of springs, scattered among the hills and near the coast [35,37]. In this area, man has been able to harmoniously integrate with the forms of landscape [38], in a rural, agricultural and pastoral civilization [5]. 

Cilento has a population of about 278,093 and maintains relatively high proportions of long-lived people (old, nonagenarians and centenarians), with a ratio of 40.31/1000 inhabitants [39]. The prevalence of centenarians remains at around the 12.49 ratio. With a substantial old population base, Cilento is an ideal region to examine whether regional longevity is associated with the properties of water elements.

### 2.2. Data Collection on Population and Water Sources

Demographic data were collected through the Cilento Municipal Archives in the years 2021–2022 and compared with ISTAT demographic statistics [5]. Longevity indicators were calculated from the collected demographic data and used as dependent variables in the study. Four longevity indicators were used in the research to reflect regional longevity [5], including the 85+ ratio (85+/total population) [40], the 90+ ratio (90+/total population) [40], and the Centenarian ratio (95+/55–64) [41,42]. To reduce the effect of important phenomena, such as declining birth rates and observed migration in regional territories, the Centenarity index (CI%), given by the ratio of centenarians to those aged 90 [43], was used (Table 1).

Physicochemical parameters of tap drinking water from the 102 municipalities in Cilento, used in the research as independent variables, were collected through CONSAC Gestioni Idriche [37] and ASIS Salernitana [44], which provide integrated water services in the Cilento area. Parameter selection was based on review of the relevant literature and availability of collected data and included: (1) hydrogen ion concentration (pH), where 7 indicates a neutral solution, a range from 0 to 7 indicates an acidic solution, and a range from 7 to 14 highlights an alkaline/basic solution [45,46,47]; (2) total hardness (TH), given by the concentration of calcium ions (Ca^2^+) and magnesium ions (Mg^2^+) [32], whose levels are as follows: 0–7 °F very soft water, 7–15 °F soft water, 15–22 °F medium-hard water, 22–35 °F hard water, >35 °F very hard water [15,45,46,47]; total dissolved solids (TDS), divided into the following levels: 50–150 excellent for drinking, 150–250 good, 250–300 fair, 300–500 poor [15]; and cations and anions, including sodium (Na^+^), nitrate (No_3_^−^), sulfate (SO_4_), chloride (Cl), iron (Fe^2+^), manganese (Mn^2+^), and nickel (Ni) (Table 2) [15,45,46,47].

### 2.3. Data Collection Procedure

In this study, first, the municipalities in Cilento with the highest concentrations of long-lived people, including old, nonagenarians, and centenarians, were identified through geomaps; second, the annual means of the most significant physicochemical, mineral and metal parameters of tap drinking water served this region were examined, evaluating data from municipalities fed by the same catchment sources. Elements with zero values were not evaluated; the parameters were then geomapped and clustered to assess their significance at the spatial level; finally, the influence that the different properties of tap drinking water had on longevity was highlighted through ordinary least squares (OLS) and geographically weight regression (GWR) models. 

### 2.4. Statistical Analysis

This study used STATA software [48] for descriptive statistics and to calculate longevity indicators based on data from the Cilento Municipal Archives 2021–2022 and the National Institute of Statistics 2022, as well as to calculate the annual means of physicochemical parameters of tap drinking water supplied by CONSAC and ASIS water service in Cilento. Before performing the statistical analysis, the Skewness and Kurtosis normality test was used to determine whether the data were normally distributed. QGIS 3.14.15 [49] was then used to produce spatial distribution maps of longevity and drinking water parameters. The global Moran’s I index was used to describe the characteristics of the global spatial cluster value of the longevity index and water parameter (for Moran’s formulas, see our previous study) [5], through ArcGIS [50]. To study the spatial relationship between the explanatory variables and the longevity outcome variables in the provided dataset, an OLS regression model and a GWR model were used and analyzed with ArcGIS 10.8 software (Esri, Redlands, CA, USA).

## 3. Results

### 3.1. Geographical Distribution of Old, Nonagenarians and Centenarians in Cilento Region

**Figure 1:**
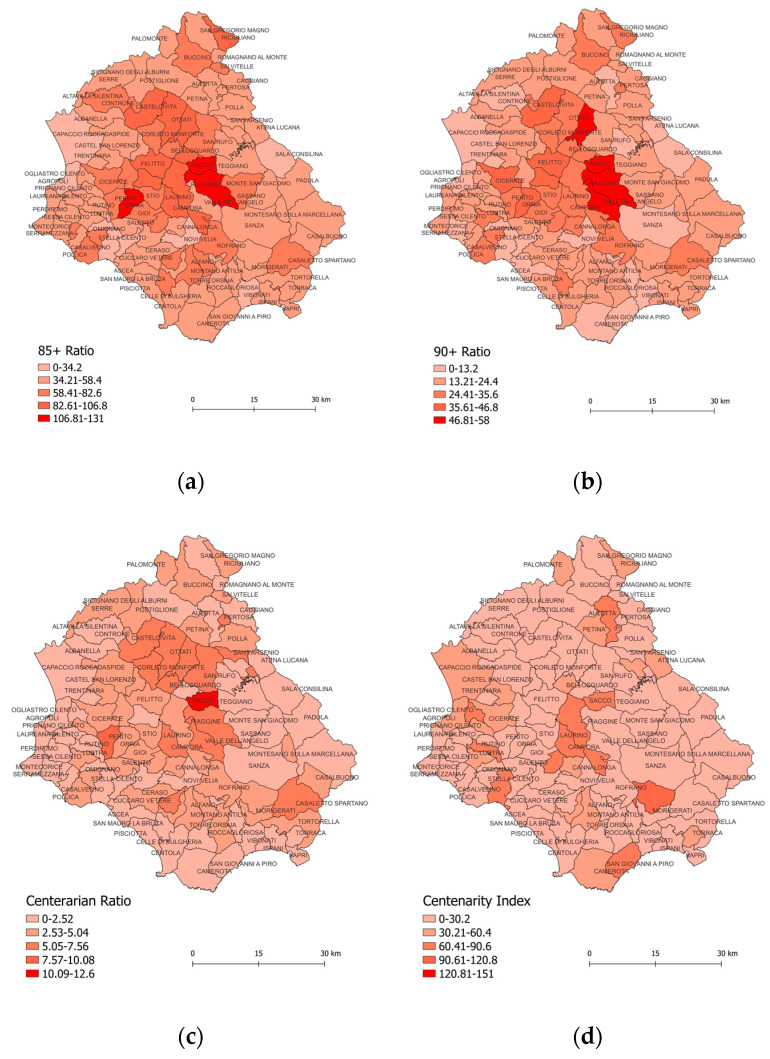
Spatial distribution of the four longevity indices in Cilento municipalities. Note: (**a**) Spatial distribution of 85+ ratio; (**b**) Spatial distribution of 90+ ratio; (**c**) Spatial distribution of centenarian ratio; (**d**) Spatial distribution of centenarity index.

Based on the new longevity population data (Table 1 and Figure 1a–d), we confirmed what was previously reported in our research [5]; namely, that the distribution of old, nonagenarians and centenarians was clearly delineated in the north–central and southeastern municipalities of Cilento.

The highest 85+ ratio was recorded in Sacco (13.1), Orria (10.9), Piaggine (10.9), Campora (10.6), and Magliano Vetere (10.3), while the lowest was recorded in Capaccio (1.0), Casal Velino (1.0), Novi Velia (2.5), and Agropoli (3.2) (Figure 1a). 

The highest 90+ ratio was recorded in Sant’Angelo a Fasanella (5.8), Sacco (5.7), Valle dell’Angelo (5.3), Piaggine (4.9), while the lowest was recorded in Capaccio (0.2), Casal Velino (0.2), Novi Velia (0.9), Castelnuovo Cilento (1) (Figure 1b). 

The highest centenarian ratio was recorded in Sacco (12.6), Perito Cilento (7.7), Pertosa (7.4), Campora (7.1), Sant’Angelo a Fasanella (7), Bellosguardo (6.7), while the lowest indices were recorded in Alfano (0), Romagnano al Monte (0), Rutino (0), Roscigno (0.1), Novi Velia (0.3), and Giungano (0.6) (Figure 1c). 

The highest CI% was recorded in Caselle in Pittari (9.3), Campora (8.3), Sacco (7.7), Moio della Civitella (7.1), while the lowest was reported for Albanella (0), Alfano (0), Casalbuono (0), Casaletto Spartano (0) (Figure 1d).

Thus, we were able to state that the longevity indicators, according to the global autocorrelation values, were spatially clustered more than would be expected if the underlying spatial processes were random, so we rejected the null hypothesis. In fact, Moran’s Z-score I values indicated spatial autocorrelation at the 0.01 significance level for the 85+ ratio and for the 90+ ratio. No significance level was found for the centenarian ratio and centenarity index values (Table 1).

### 3.2. Trace Elements Concentration in Tap Drinking Water

**Figure 2:**
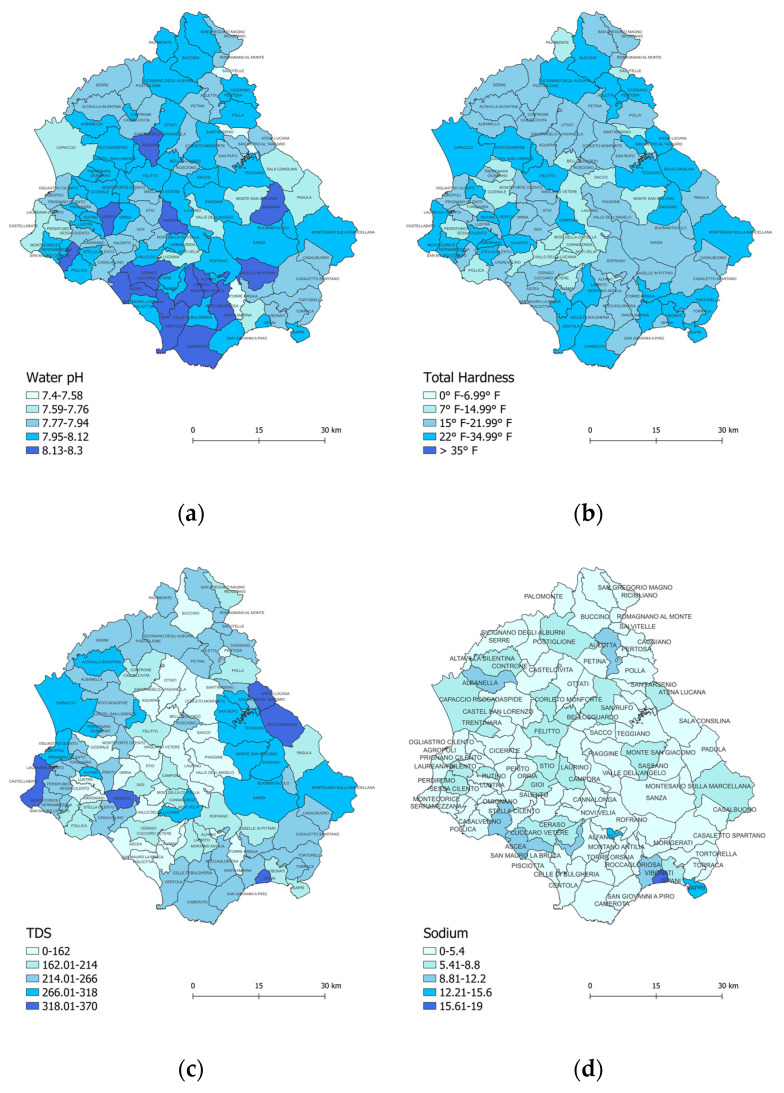

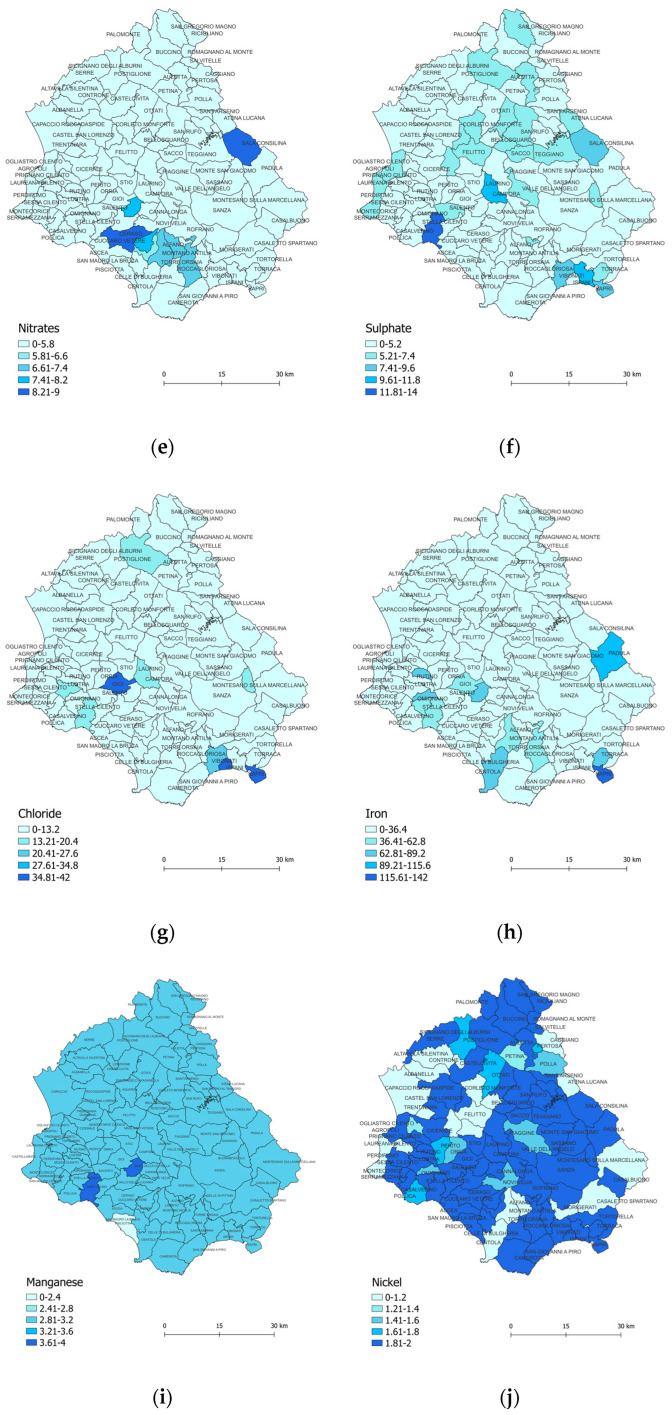
Spatial distribution by Cilento municipalities of tap drinking water elements. Note: (**a**) Spatial distribution of water pH; (**b**) Spatial distribution of total hardness TH; (**c**) Spatial distribution of total dissolved solids TDS; (**d**) Spatial distribution of sodium; (**e**) Spatial distribution of nitrates; (**f**) Spatial distribution of sulphate; (**g**) Spatial distribution of chloride; (**h**) Spatial distribution of iron; (**i**) Spatial distribution of manganese; (**j**) Spatial distribution of nickel.

The descriptive statistics and spatial distribution of the tap water elements of the Cilento municipalities are shown in Table 2 and Figure 2a–j, respectively.

The tap drinking water in the 102 municipalities of Cilento falls within the WHO definition of fresh water [15].

First of all, the water pH values in the various Cilento municipalities ranged from 7.4 to 8.3, which meant that the drinking water of this Southern region of Italy was all alkaline, and differentiated by the weakly to strongly alkaline level (Figure 2a). The municipalities with a higher concentration of long-lived people had an alkalinity level between 7.9 and 8.2.

The TH (given by the concentration of Ca^2^+ and Mg^2^+) [32] of tap drinking water ranged from 9.6 °F to 34.8 °F, which means that the water varied between soft water (TH = 7–15 °F) and hard water (TH = 25–35 °F) (Figure 2b). 

The TDS showed differences in levels among the 102 Cilento municipalities, with drinking water values ranging from excellent to poor (Figure 2c). 

The concentrations of Na^+^, No_3−_, So_4_ and Cl_ showed minimal spatial differences among the 102 Cilento municipalities (Figure 2d–g), and, as for heavy metals, no traces of arsenic, lead, mercury, cadmium were detected; only Ni was evident, with a minimum concentration of 1.7 μg/L out of the 20 μg/L recommended by National Standard (NS) and 70 μg/L recommended by World Health Organization guidelines (WHOGV) (Figure 2j). Copper was nil, while Mn had a mean concentration of 3 μg/L compared with the 50 μg/L recommended by NS and 400 μg/L established by WHOGV (Figure 2i). The highest concentrations of iron were found in areas not inhabited by the long-lived (Figure 2h). The concentrations of all elements were well below the recommended standards (Table 2). In fact, tap water quality in Cilento municipalities was evaluated according to recommended standards, both national [45,46] and World Health Organization [15]. As a result of this assessment, all drinking water in Cilento can be considered of good quality.

In addition, Moran’s I index was used to test for the presence of spatial autocorrelation and to identify whether case densities were randomly distributed in the study area. We found that longevity densities were clustered according to certain elements of tap drinking water, such as water pH (*p* = 0.004), TDS (*p* = 0.003), iron (*p* = 0.02), nitrate (*p* < 0.001), chloride (*p* = 0.001).

### 3.3. Relationship between Tap Drinking Water Elements and Longevity

**Figure 3:**
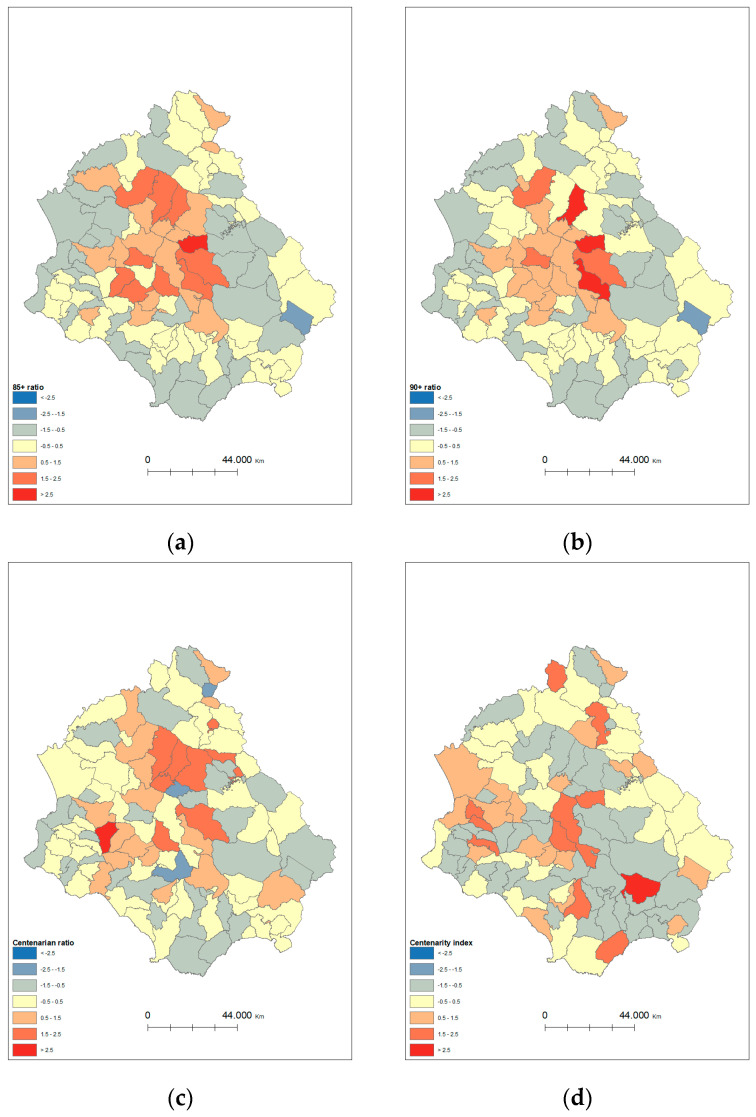
Results of the GWR models for 85+ ratio, 90+ ratio, centenarian ratio and centenarity index showing the local R2. Note: (**a**) local R2 for 85+ ratio; (**b**) local R2 for 90+ ratio; (**c**) local R2 for centenarian ratio; (**d**) local R2 for centenarity index. GWR—geographically weight regression.

OLS and GWR regression models were used to evaluate the relationship between longevity and tap water elements (Table 3 and Figure 3a–j, respectively). OLS regression results showed a negative relationship with TH, TDS, sodium and iron for both the 85+ ratio and 90+ ratio. In addition, a negative relationship was found with nickel for the 85+ ratio, while a positive relationship was found with chloride for the 90+ ratio. The centenarian ratio also had a negative relationship with TH, TDS, sodium and iron, and a positive association with chloride. Finally, the centenarity index showed a positive correlation with manganese.

For the GWR models, shown in Figure 3a–j; all OLS regression variables were used.

## 4. Discussion

In the present study, the relationship between the physicochemical elements of drinking water and longevity in Cilento was investigated. Based on the results, several elements emerged to support our hypothesis that drinking water in Cilento region can affect health and longevity.

Water is essential for hydration and, thus, for life; in fact, in the human body, thanks to its special properties, numerous vital functions can take place regularly and provide benefits [13]. The main purpose of a drinking water supply is to protect human health, to restore lost health to those suffering from conditions such as hypertension, diabetes and other chronic diseases, and to prevent dehydration, which is associated with increased disability in the old [51,52].

The beneficial properties of water have also been highlighted by studies conducted on the long-lived Hunza people [53,54], whose longevity was due not only to their simple and healthy lifestyle, but also to the use of water with a high pH level. Cilento, as our results showed, was characterized by both the presence of nonagenarians and centenarians, and by the presence of alkaline drinking water in all 102 municipalities. Indeed, what differentiates the municipalities was the weakly to strongly alkaline level of drinking water. Municipalities with a higher concentration of long-lived people had a higher alkalinity level, ranging from 7.9 to 8.2, than those without long-lived people (ranging from 7.4 to 7.8). In addition, the Moran I index showed that longevity density is significantly correlated with water pH. This result is in agreement with Jin et al. [27], who confirmed that hydrogen-rich functional water has been introduced as a therapeutic strategy for health promotion and disease prevention. Other studies in animal models have shown positive effects, improving body weight and offspring development [25,26] with the use of alkaline water. In addition, several biological effects have been documented, such as antidiabetic and antioxidant actions [27], DNA-protective effects [28], and growth-promoting activities [26]. Further confirmations of our findings come from Magro et al., who conducted a 3 year survival study on 150 mice, in which the biological effects of alkaline water consumption were investigated in depth. The results showed that the mice experienced benefits in longevity in terms of the “aging deceleration factor” [55].

An important etiological factor worldwide is hard water (sum of Ca^2+^ and Mg^2+^), which causes many diseases such as cardiovascular problems, diabetes, kidney dysfunction, and neural diseases. According to our results, water varied between soft and medium-hard in the Cilento municipalities that hosted the long-lived groups; the regression outcome showed a significant inverse relationship between the long-lived (85+ ratio, 90+ ratio, Centenarian ratio) and hard water. In agreement with our results, many studies have reported an inverse relationship between drinking water hardness and cardiovascular disease [12,56,57,58,59], or a weak inverse relationship between risk factors such as hypertension, smoking habit, and elevated serum lipids [60,61].

Regarding the statistically significant results for Total Dissolved Solids, which refer to the total dissolved substances in drinking water that confer a bitter or salty taste to the water, the values of tap drinking water varied from excellent to poor, with differences in levels among the 102 municipalities in Cilento. Municipalities with the lowest TDS, such as Sacco, Cuccaro Vetere, Pertosa, San’Angelo a Fasanella, Bellosguardo, Piaggine, and others, had excellent water levels and higher concentrations of long-lived people.

Drinking water is a major contributor to daily sodium intake, but in areas where there are large concentrations of salinity, this can become a risk factor for hypertension [62]. In Cilento, the highest sodium concentrations were found mainly in the northwestern and southwestern areas, while the central areas, where the long-lived were concentrated, had lower amounts of sodium. Low-sodium drinking water sources in Cilento could help prevent hypertension-related morbidity and mortality for the large number of people living in these areas, evidence that is in line with the significant inverse results of our regression. Moreover, these results agreed with the study of Sacks et al. [63], in which it was reported that with the reduction in sodium intake from high (3.6 g/day) to intermediate (2.3 g/day) in the control group, systolic blood pressure decreased by 2.1 mm Hg, and further reductions in sodium intake from intermediate to low led to a further reduction in blood pressure of 4.6 mm Hg. Systematic reviews and meta-analyses of randomized controlled trial (RCT) studies have reported links between high sodium intake and increased blood pressure in the population [64,65,66]. Excessive sodium intake is also directly associated with stroke [67] and elevated levels of inflammatory markers [68,69]. Reduced sodium intake is associated with improved renal function, reduced arterial stiffness, and vasodilation of small blood vessels [70].

Iron was present with consistent values throughout Cilento, with a higher value toward the southwestern coastal area outside the longevity area, and was significantly clustered and inversely correlated with local longevity (85+ ratio, 90+ ratio, and centenarian ratio). These results indicate that iron is an essential trace element for life, but can generate toxicity under overloaded conditions. According to Baschant et al. [71], the accumulation of iron in organs and tissues, if untreated or poorly controlled, can lead to complications such as liver cirrhosis, heart failure or diabetes.

In Cilento, with regard to heavy metals in the water, no traces of arsenic, lead, mercury, cadmium, and copper were detected, but only nickel was evident, with a minimum concentration of 1.7 μg/L, a parameter well below the values recommended not only by NS and WHOGV but also by the European Food Safety Authority, which established a tolerable daily intake (TDI) of 13 μg/L body weight. According to data reported in the United States on tap drinking water, nickel concentrations ranged from 0.55 to 25 μg/L; in Australia, nickel concentrations in drinking water were generally <10 μg/L [72], and, according to European Food Safety Authority (EFSA), which evaluated the results of several European surveys on nickel in drinking water, the mean lower and upper limits for all samples was 2 and 3 μg/L, respectively [73]. These data confirm that the value of nickel in Cilento tap drinking water is lower than the concentrations reported in other nations and does not pose a health risk.

Of note, a trend of positive association was observed between manganese in Cilento tap drinking water and the centenarity index. This result is in agreement with the US National Research Council (NRC), which has established a safe and adequate dietary intake (ESADDI) of 2–5 mg/day of Mn for adults [74]. Furthermore, Mn in humans is known to vary with life stage and sex [75], becoming essential during old age. Herein, Mn is an essential nutrient required for a number of metabolic functions, including those involved in energy metabolism, immune system improvement, nervous system function, blood coagulation and the activity of antioxidant enzymes that protect cells from free radical damage [76].

On the basis of the data, this study has some limitations. First, longevity is the result of a combination of genes, the natural environment and socioeconomic status. Therefore, other factors could interfere with the relationship between the water environment and longevity. Second, it is not known whether the conclusions obtained in the Cilento region can be applied to other longevity areas, due to their different geological conditions, climate, etc. Therefore, it is of great importance to study the effects of tap drinking water on longevity based on the common characteristics of drinking water quality in multiple longevity areas. Furthermore, we did not evaluate the influence of Cilento tap water on common diseases in the region. In addition, the water monitoring data provided by the Cilento Water Service Company lacked some trace elements, such as potassium, zinc, and individual calcium and magnesium data, as we only had data on overall water hardness (sum of Ca^2^+ and Mg^2^+).

Further research will be needed in the future to establish a holistic assessment between different areas of longevity in Cilento, tap water quality and common diseases in the region, including thorough in vivo research in mice.

## 5. Conclusions

In conclusion, we can say, to the best of our knowledge, that this is the first study to investigate the possible relationship between longevity in Cilento and the elements present in tap water at the municipal level. The results of the study confirmed that there is a high prevalence of old, nonagenarians and centenarians in Cilento, concentrated in the north-central and southeastern areas of the territory, and that some elements in tap drinking water may contribute to local longevity. In particular, drinking water in the 102 Cilento municipalities was found to be alkaline and falls within the WHO definition of fresh water. Regarding heavy metals, no traces of arsenic, lead, mercury, cadmium and copper were detected, but only minimal concentrations of nickel, iron and manganese, well below the NS and WHOGV recommended values. The municipalities with higher longevity concentrations had higher alkalinity levels than the other municipalities, soft to medium-hard water hardness, total dissolved solids equivalent to excellent water levels, lower sodium concentrations, advantageous iron concentration, and an adequate dietary intake of manganese per day.

## Figures and Tables

**Table 1:** Descriptive characteristics of longevity indicators among the 102 Cilento municipalities.

Indicators	Number of Municipalities	Min	Max	Mean	SD	Moran’s I Index	Z-Score	*p* Value
85+ ratio	102	1	13.1	5.6	2.2	0.37	6.18	<0.001 **
90+ ratio	102	0.2	5.8	2.1	1.04	0.30	5.04	<0.001 **
Centenarian ratio	102	0	12.6	3.08	2.01	0.04	0.93	0.34
Centenarity index	102	0	10	1.9	2.6	−0.03	−0.33	0.73

Note: Min—minimum; Max—maximum; SD—standard deviation; ** denotes *p* > 0.01, indicates the statistical significance of the Moran’s test; z-score indicate statistical significance of clustering (it must be greater than 2.58 at *p* < 0.01; *p* value probability of error in rejecting the null hypothesis).

**Table 2:** Descriptive statistics of the level of element concentration in tap water in Cilento and Global Moran’ s I values.

Indicators	Number of Municipalities	Min	Max	Mean	SD	NS	WHO GV	Moran’s I Index	Z-Score	*p* Value
pH	102	7.4	8.3	7.9	0.2	6.5–9.5	6.5–8.5	0.16	2.85	0.004 *
TH	102	9.6	34.8	19.7	4.8	15–50	15–50	−0.07	−1.34	0.17
TDS	102	110	370	219.6	58.1	1000	1000	0.21	3.57	0.003*
Na^+^	102	2	19	5.2	2.7	200	200	0.02	0.61	0.53
No_3−_	102	5	9	5.2	0.7	50	50	0.28	5.08	<0.001 *
So_4_	102	3	14	4.5	1.8	250	250	0.07	1.39	0.16
Cl_	102	6	42	9.9	4.8	250	250	0.16	3.20	0.001 *
Fe^2^+	102	10	142	21	22.4	200	200	0.12	2.29	0.02 **
Mn^2^+	102	<3	<3	3	0.1	50	400	−0.004	0.10	0.92
Ni	102	1	2	1.7	0.4	20	70	0.03	0.74	0.45

Note: pH—hydrogen ion concentration; TH—total hardness (given by the concentration of calcium ions (Ca^2^+) and magnesium ions (Mg^2^+)); Na^+^—sodium; No_3−_—nitrate; So_4_—sulphate; Cl_—chloride; Fe^2^+—iron; Mn^2^+—manganese; Ni—nickel; NS—National Standard; WHO GV—World Health Organization guidelines; * denotes *p* < 0.05; ** denotes *p* < 0.01, indicates the statistical significance of the Moran’s test; z-score indicate statistical significance of clustering (it must be greater than 1.96 at *p* < 0.05 and greater than 2.58 at *p* < 0.01); *p* value probability of error in rejecting the null hypothesis.

**Table 3:** Results of OLS regression.

Longevity Indicators	Tap Drinking Water Elements	Coefficient	t	*p* Value
Model 1 (R^2^ 0.32 AICc 906.91) 85+ ratio	Water pH TH TDS Sodium Nitrates Sulphate Chloride Iron Manganese Nickel	2.76 −4.83 −0.22 −1.77 −1.15 2.23 0.53 −0.22 −7.71 −7.56	0.24 −2.81 −6.33 −1.90 −0.46 1.86 0.95 −2.60 −0.65 −1.54	0.79 0.005 ** <0.001 ** 0.03* 0.50 0.17 0.28 0.002 ** 0.51 0.04 *
Model 2 (R^2^ 0.37 AICc 754.28) 90+ ratio	Water pH TH TDS Sodium Nitrates Sulphate Chloride Iron Manganese Nickel	9.50 −1.79 −0.10 −1.34 −0.95 0.83 0.54 −0.09 0.71 −2.30	1.58 −1.86 −6.14 −3.04 −0.80 1.45 2.05 −2.24 0.12 −0.98	0.11 0.004 ** <0.001 ** 0.002 ** 0.42 0.26 0.02 * 0.02 * 0.93 0.32
Model 3 (R^2^ 0.25 AICc 407.77) Centenarian ratio	Water pH TH TDS Sodium Nitrates Sulphate Chloride Iron Manganese Nickel	−0.38 −0.23 −0.01 −0.21 0.20 0.13 0.10 −0.02 0.83 −0.67	−0.38 −1.52 −4.33 −2.61 0.92 1.28 2.04 −2.69 0.80 −1.55	0.70 0.009 ** 0.009 ** 0.002 ** 0.35 0.20 0.005 ** 0.004 ** 0.42 0.12
Model 4 (R^2^ 0.15 AICc 960.91) Centenarity index	Water pH TH TDS Sodium Nitrates Sulphate Chloride Iron Manganese Nickel	16.63 −1.74 −0.02 −1.09 −2.67 1.59 0.39 −0.06 50.01 2.30	1.13 −0.78 −0.57 −0.91 −0.36 1.02 0.54 −0.54 3.27 0.36	0.26 0.43 0.51 0.36 0.71 0.30 0.58 0.58 0.001 ** 0.71

Note: * significant *p* value ≤ 0.05; ** significant *p* value ≤ 0.01; Model 1–4 indicate the models analyzed with OLS; R^2^ indicates R-squared, coefficient of determination; AICc—Akaike’s Information Criterion; TH-total hardness; TDS—total dissolved solids; OLS—ordinary least squares.

## Data Availability

Data included in this manuscript were provided by the National Institute of Statistics and the Cilento Municipal Archives for populations; and by the official Integrated Water Service Providers in the Cilento area for water. Therefore, we are not authorized to share the data with third party organizations. However, the corresponding author is available to provide any explanation to the Editor if requested.
